# *In vivo* effect of an luteinizing hormone-releasing hormone analog on vascular endothelial growth factor and epidermal growth factor receptor expression in mammary tumors

**DOI:** 10.4103/1477-3163.51852

**Published:** 2009-06-02

**Authors:** Ana Isabel Flores, Fernando Bedoya, Montserrat Grau, Rafael Enríquez de Salamanca, Irene Vegh

**Affiliations:** Centro de Investigación, Hospital Universitario 12 de Octubre, Av. Córdoba s/n, CP28041, Madrid, Spain

**Keywords:** Epidermal growth factor receptor, luteinizing hormone-releasing hormone, mammary tumors, vascular endothelial growth factor

## Abstract

**Background:**

The hypothalamic luteinizing hormone-releasing hormone (LHRH) is well known for its role in the control of pituitary gonadotropin secretion and it has demonstrated a direct antiproliferative effect on some cancer cell lines of LHRH and its synthetic analogs. The study was designed to assess whether administration of the LHRH analog (goserelin) has any effect on the expression of the vascular endothelial growth factor (VEGF) and the epidermal growth factor receptor (EGFR) in rats with N-nitroso-N-methylurea (NMU)-induced-mammary tumors “*in vivo*”

**Materials and Methods:**

The animals with tumors were assessed after acute or chronic treatment with goserelin, and in all the animals VEGF and EGFR expression was examined both in plasma and tumor homogenates by enzyme immunoassay.

**Results:**

The basal plasma values of VEGF were lower in the healthy control group than in rats with NMU-induced tumors (*P* = 0.025). Following acute treatment with goserelin, VEGF expression in plasma increased above basal levels after 60 min (*P* = 0.05) and dropped during chronic treatment. Likewise, in the tumor homogenate the mean VEGF expression was higher at 60 min post-goserelin administration than the basal levels, although VEGF expression then diminished at 90 min. Plasma EGFR expression was higher in rats with NMU-induced tumors than in healthy controls (*P*<0.01).

**Conclusions:**

The results allow us to conclude that goserelin may exert a short-term stimulatory effect on the release of VEGF, as well as a long-term inhibitory effect on VEGF but not EGFR expression.

## INTRODUCTION

As well as contributing to numerous normal and pathological conditions, angiogenesis is an essential determinant in the development of tumors and metastasis. In this regard, the over-expression of the vascular endothelial growth factor (VEGF) has received much attention due to its role in angiogenesis and since it was detected in different malignant tumors, including both human and experimentally induced tumors. Indeed, VEGF has been associated with tumor growth[1] and it has been shown to be a potent vascular permeability factor, a vascular endothelial cell mitogen and a survival factor.[2] VEGF over-expression is stimulated by hypoxia and in this pathway; the hypoxia inducible growth factor-1 (HIF-1) promotes VEGF expression and angiogenesis.[3–5] Moreover, over-expression of VEGF and/or its receptor is associated with an unfavorable prognosis and contributes to the progressive growth of mammary cancer. In experimental studies, VEGF seems to only be essential for the early stages of breast cancer development.[6] The contribution of VEGF to tumor evolution appears to occur through both paracrine and autocrine mechanisms, as seen in transgenic mice.[7] Indeed, very little is currently known about how other micro-environmental factors may contribute to angiogenesis in mammary tumors.

When mammary carcinomas are induced in Sprague-Dawley rats with N-methyl-N-nitrosourea (NMU), 73% of the animals develop tumors.[8] However, once the tumor is established, castration arrests tumor growth or causes a temporary regression of the tumor, indicating a degree of hormone dependency. Similarly, NMU induced mammary tumor metastases in bone marrow and spleen, and the tumors induced by this carcinogen, are also transplantable.[8]

Gonadal function is regulated by the gonadotrophin follicle-stimulating hormone (FSH) and luteinizing hormone (LH) released from the pituitary gland. This secretion is in turn controlled by gonadotrophin releasing hormone (GnRH). Since its identification,[9] different potent GnRH agonists have been developed, including goserelin acetate.[10] The efficacy of GnRH agonists was confirmed in early-stage breast cancer as adjuvant therapy[11] and as a therapy for prostate carcinoma. More recently, we showed that goserelin administration produces the regression of NMU-induced mammary tumors through the inhibition of prolactin (PRL), tumor necrosis factor (TNF)-alpha and nitric oxide expression.[12]

Mammary tumor growth partially depends on complex interactions between different growth factors and their corresponding receptors and inhibitors. The epidermal growth factor (EGF) family and the expression of their receptor (EGFR) were found to be correlated with the grade and localization of neoplastic tumors.[13] EGF is one of the many growth factors that may drive VEGF expression and vascular endothelial growth factor receptor-1 (VEGFR-1) may help human cancer cells resist EGFR inhibitors.[14] Indeed, EGFR inhibitors decrease VEGF expression by down-regulating the PI3K/Akt pathway that in turn diminishes VEGF promoter activity through two different pathways, one involving Sp1 and another involving the hypoxia inducible factor-1 alpha (HIF-1alpha).

Several studies have demonstrated that EGFR is over-expressed in tumors and that it is associated with the appearance of tumor cells in lymph nodes in different human neoplasias. EGFR is often over-expressed in breast and other cancers, and it is correlated with a poor prognosis. These observations have led to the development of different inhibitors that are associated with the different mechanisms to inhibit the EGFR. However, it has been proposed that there is no association between EGFR expression and the poor outcome in colorectal cancer.[15–17] Here, we have examined VEGF and EGFR expression in plasma and tumor homogenates of mammary adenocarcinomas in order to evaluate the effect of goserelin administration, the potent LHRH agonist, “*in vivo*”.

## MATERIALS AND METHODS

### Animals, protocols, and experimental design

Female Wistar rats were fed on a standard diet and allowed free access to water, during a 16h night: 8h day photoperiod at 20–22°C. All studies began when the animals were 45 days old (weight 200–240 g), and they were carried out in accordance with the Royal Decree 223/1988 (BOE 8, 18) and the Ministerial Order of 13^th^ October 1989 (BOE 8) regarding the protection of experimental animals, as well as with the European Council Directive 86/609/EEC.

In this study, the rats were first divided into two groups:

**Group 1** - The control (n = 10) basal healthy (BH) rats not exposed to NMU administration.

**Group 2** - Rats with NMU-induced tumors (n = 27). NMU tumors were induced in 45-day-old rats according to our previous protocol.[14] This latter group was then subdivided into 3 further groups (A, B and C):

- Group A control basal tumor (BT) group (n = 7), was set up with sham intravenous jugular injections, with vehicle alone.
- Group B goserelin acute treatment (n = 10) received goserelin i.v. “in bolus” 0.25 mg/ml/rat. In these animals, basal samples were taken prior to goserelin administration (BT) and at 30, 60, and 90 minutes after the injection of the drug.
- Group C (n = 10) received chronic subcutaneous administration of goserelin (0.25 mg/ml/day) over 60 days.

Rats received the goserelin (acute and chronic treatment) when the size of tumor was 1.5 cm diameter.

The rats were sacrificed at the end of the experiments and all tumors were examined histologically after death to confirm that they were all adenocarcinomas.

### Chemicals

Crystalline NMU (Sigma, No. N1517) was dissolved in 0.85% NaCl solution and acidified with acetic acid at 5.0 pH. The concentration was then adjusted to 5 mg/100 g body wt/rat (final volume of 1 ml) which was administered intraperitoneally.

The pure goserelin acetate LHRH analog ([D-Ser (Bu^t^)^6^-Aza-Gly^10^-GnRH]) was obtained from Astra-Zeneca, UK. The rat VEGF assay was performed on plasma and the tumor homogenate was analyzed using a rat-VEGF enzyme immune-assay (ELISA, catalog number RRVOO, R and D Systems, Barcelona, Spain) according to the manufacturers' instructions. VEGF plasma values were expressed as pg/ml, and in tumor homogenates, as pg/mg of the total protein concentration. The EGFR assay was performed on plasma and on a homogenized tumor supernatant using an EIA EGFR kit (BLK Diagnostics, Barcelona). The buffer A used contained: 1 mM dithioerythritol, 0.01% sodium azide, 1 mM MgCl_2_, 250 mg sucrose, 10 mM sodium molybdate in 20 mM Tris-HCl (pH 7.8).

### Tumor detection

After intraperitoneal injection of NMU the animals were examined for tumor masses twice-weekly post-carcinogen administration by palpation. The size of any tumors was measured with calipers and their histological parameters were examined.

### Histology

Mammary gland tumors were diagnosed by histological examination of paraffin sections stained with hematoxylin/eosin. A mammary cancer nodule was designated as an active tumor and the number of active tumors was expressed per rat with mammary tumors.

### Preparation of blood samples and tumor fraction

Blood samples were prepared from heparin-treated blood (0.2 ml) at the times established, and they were obtained by jugular vein puncture from rats lightly anesthetized with ether. Part of the tumor was also removed immediately after each blood sample was obtained. At the end of the study the animals were sacrificed by decapitation and all further steps were performed at 0–4°C.

#### Tumor homogenization and fraction collection

After washing in ice-cold PBS (pH 7.4), 100 to 200 mg of tumor tissue was cut into small portions (the necrotic areas having been previously removed) and it was homogenized in buffer A using a glass-glass homogenizer in a weight-to-volume ratio of 1 to 3. The homogenate was isolated by centrifugation at 106,000xg for 30 min and the supernatants were stored in aliquots at −80°C until use. The total protein concentration was determined by the Lowry assay using a commercially available kit in accordance with the manufacturer's instructions (Bio-Rad, Madrid, Spain).

### VEGF determination in plasma and in tumor supernatant

The 96-well ELISA assay used was a quantitative sandwich enzyme immunoassay using a monoclonal antibody specific for rat VEGF pre-coated on a microplate. Standards, controls and samples (50 *μ*l each) were pipetted into the wells and the VEGF present was bound by the immobilized antibody. Previously, 50 *μ*l of assay diluent was added to each well and incubated for 2 hours at room temperature on a shaker. After washing five times with 400 *μ*l of wash buffer per well, 100 *μ*l of a horseradish peroxidase conjugated polyclonal antiserum against rat VEGF was added to the wells and incubated for 1 hour at room temperature on the shaker. Unbound material was removed from the wells by washing five times, and 100 *μ*l of a substrate solution was added to the well and incubated for 20 minutes at room temperature in the dark. The reaction was stopped with 50 *μ*l of stop solution (hydrochloric acid) and the intensity of the color precipitate measured was proportional to the amount of VEGF initially bound. The VEGF concentration in tumor supernatant was expressed with respect to total protein (pg/mg protein).

### EGFR in the plasma and tumor supernatant

The ELISA assay for EGFR expression in plasma and tumor membranes was performed according to the manufacturers' instructions. The tumor membrane pellet was re-suspended in buffer A and centrifuged at 1500 rpm for 5 minutes to obtain the cell membrane fraction for EGFR determination. EGFR levels were expressed as fmol/mg membrane protein.

### Calculation of results

To determine optical densities, the absorbance at 450 nm was obtained in an ELISA reader using MULTISKAN EX (Thermo electron Corporation, Lab-Center SL, Madrid Spain). After the optical density of each well was obtained (standards and samples), a standard curve was constructed by plotting the mean absorbance for each standard on the y-axis against the concentration on the x-axis, and a best fit curve was drawn through the points on the graph. The best-fit line was determined by regression analysis.

### Assay Specificity and Sensitivity

The VEGF assay recognizes both recombinant and natural rat VEGF at a minimum detectable dose of 3.9–25.0 pg/ml. There was no detectable cross reactivity with any of the proteins tested in the EGFR assay and the sensitivity of this assay was less than 0.078 fmol/ml, (mean plus three standard deviations).

### Statistical Analysis

The differences in tumor incidence were demonstrated by the x^2^ test with the Yates correction. Data were expressed as the means ± SEM and all data were assayed by duplicate. Inter-group comparisons were performed with the Student's t test. Values of *P* < 0.05 were considered to be significant.

## RESULTS

### Tumor incidence

After the 2^nd^ administration of NMU, mammary cancer was detected on the 90^th^ – 120^th^ day in 27 of the 30 (90%) rats. Indeed, the incidence of tumors per rat was 1.5 tumors. The 3 rats that didn't develop tumors after carcinogen exposure were not studied for goserelin administration. There was no microscopic tumor in these rats that did not develop tumors.

### Histological characterization of mammary tumors

**Figure 1:**
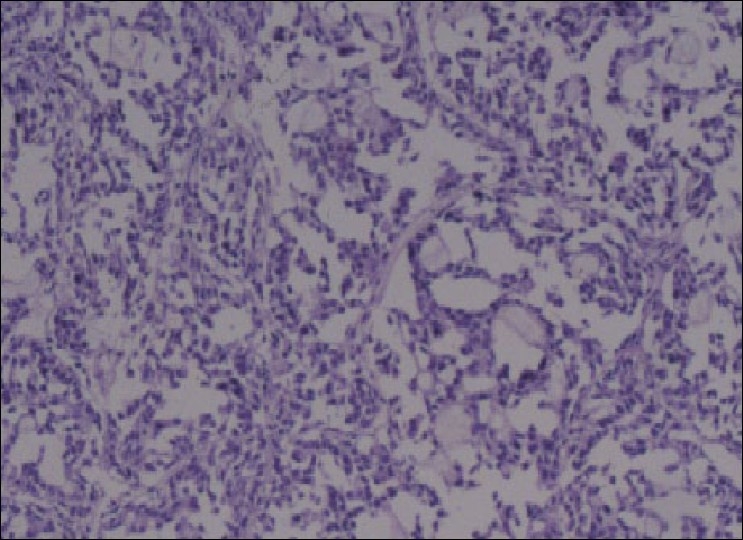
Histological characterization of NMU-induced mammary tumors in Wistar rats. Hematoxylin-eosin stained tissue section showing the typical histological appearance of solid mammary adenocarcinoma from one of the NMU induced rats (HandE, 200×).

The histological study revealed that all the tumors studied were adenocarcinomas [Figure 1]. In terms of size, the tumors in the rats suffered an important involution or remission, and the tumors were reduced in size by more than 65% after 60 days of goserelin treatment. In the central areas of the majority of tumors, necroses could be observed macroscopically.

### Expression of VEGF in the plasma of control and NMU-treated rats

**Figure 2:**
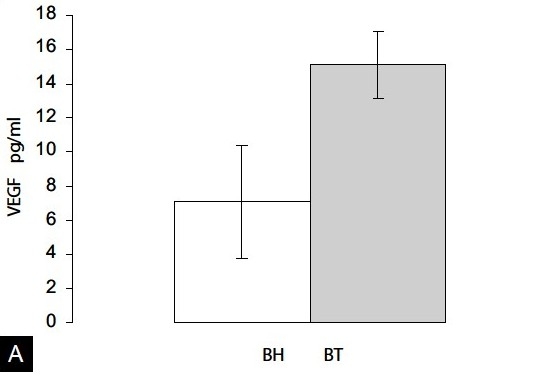

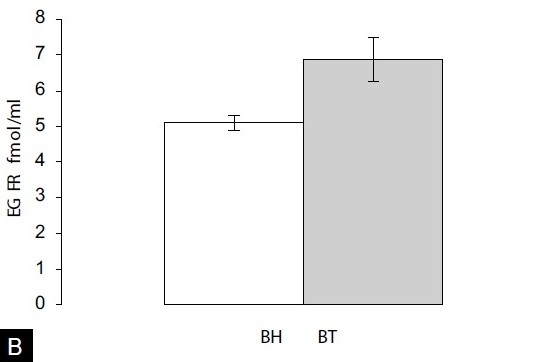
VEGF and EGFR expression in plasma from basal healthy (BH) and basal NMU induced tumor (BT) rats. (A) In the BT animals the mean VEGF expression was higher than in rats without tumors BH (*P* < 0.025, values expressed as pg/ml). (B) The mean EGFR expression was higher in rats with induced tumors than in healthy rats (*P* < 0.01, values expressed as fmol/ml).

In the healthy control group that did not develop NMU-induced tumors, the mean basal levels (BH) of circulating VEGF were 7.1 ± 3.3 pg/ml (n = 10, mean ± SEM). By contrast, in the animals with NMU-induced tumors the basal levels of VEGF expression (BT) were 15.1 ± 1.9 pg/ml (n = 7). Thus, it was clear that the mean VEGF expression was higher in the group of rats with NMU-induced tumors than in the healthy rats (*P* < 0.025, Figure 2 A).

Following the acute (bolus) treatment with goserelin (n = 10), the plasma levels of VEGF initially rose from the basal levels to 21.5±1.3 pg/ml (*P* = 0.02) at 30 min and 20.7±1.6 pg/ml (*P* = 0.05) at 60 min, before falling to 15.3±8.1pg/ml at 90 min (*P* = 0.97).

**Figure 3:**
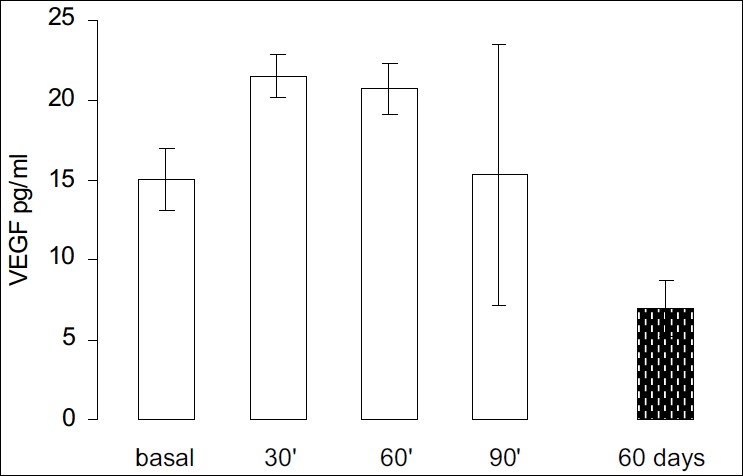
Time course of plasma VEGF expression after goserelin administration “in bolus” and chronic exposure. VEGF expression increased with respect to the basal (BT) values at 30 min (*P* = 0.02) and 60 min (*P* = 0.05), and decreased at 90 minutes (*P* = 0.97). Chronic goserelin administration led to a fall in the mean VEGF levels to basal pre-treatment levels. The values were expressed as pg/ml and each bar represents the mean ± SEM.

In animals exposed to chronic (60 days) goserelin treatment the mean VEGF values in plasma were similar to those in the healthy controls (BH) without tumors (7.0±1.7 pg/ml, n = 10, Figure 3) and lower than basal values (BT) with tumors.

### Expression of VEGF in the tumor supernatant of NMU induced rats

The basal VEGF expression was 1,020.1±371.5 pg/mg protein (mean ± SEM, n = 10) and while there was an increase in VEGF in the tumors at both 30 min (1,232.6±705.2 pg/mg, *P* = 0.81) and 60 min (5,474.4±2,947.9 pg/mg, *P* = 0.05) after goserelin administration, the levels of VEGF fell sharply after 90 min when compared to the basal levels (144.6 ± 68.9 pg/mg, *P* = 0.09).

**Figure 4:**
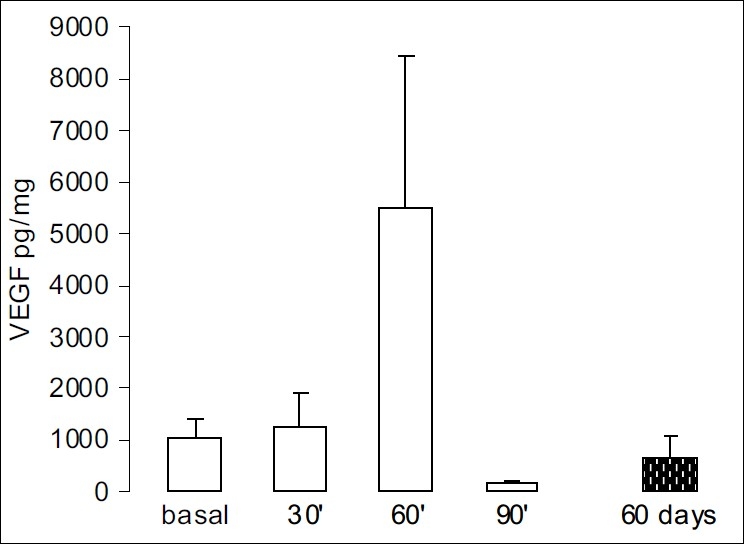
Time course of VEGF expression after goserelin administration (“in bolus”) in the tumor supernatant: BT (basal) versus 30 min, *P* = 0.81; BT versus 60 min, *P* = 0.05; and BT versus 90 minutes, *P* = 0.09. After a 60-day administration of goserelin the VEGF expression was similar to the basal values. The values were expressed as pg/mg protein and each bar represents the mean ± SEM.

Chronic treatment (60 days) with goserelin also appeared to produce a drop in VEGF expression in the tumors (632.6±446.8 pg/mg protein, n = 10) although when compared to the mean basal values, this difference was not statistically significant (*P* = 0.25, Figure 4).

## EGFR expression in rats with NMU-induced tumors

### In plasma

The basal EGFR expression in healthy control rats (BH, n = 10) was 5.1±0.2 fmol/ml (mean ± SEM) and it increased in tumor-induced rats (BT, n = 7) to 6.9±0.6 fmol/ml (*P* < 0.01, Figure 2B).

**Figure 5:**
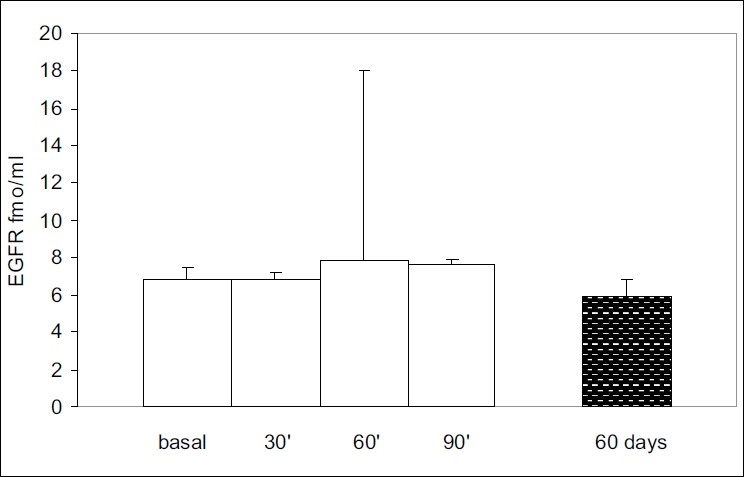
EGFR expression in plasma did not present statistically significant variations either following acute (“in bolus”) administration or during chronic goserelin treatment. The values were expressed as fmol/ml and each bar represents the mean ± SEM. BH basal healthy and BT basal values in rats with NMUinduced tumors.

Acute (bolus) treatment with goserelin did not produce any change in EGFR expression in the plasma after 30 min when compared to the basal (BT) levels (6.9±0.3 fmol/ml, *P* = 0.45). Indeed, although EGFR expression appeared to have increased at 60 (7.8±10.2 fmol/ml) and 90 min (7.6±0.3 fmol/ml) after goserelin administration, these levels were not significantly different from the basal values (BT, *P* = 0.15). Likewise, the EGFR expression after 60 days of chronic goserelin treatment (5.9±1 fmol/ml, n = 10) was not significantly lower than the basal (BT) expression (*P* = 0.20, Figure 5).

### In the tumor membrane

**Figure 6:**
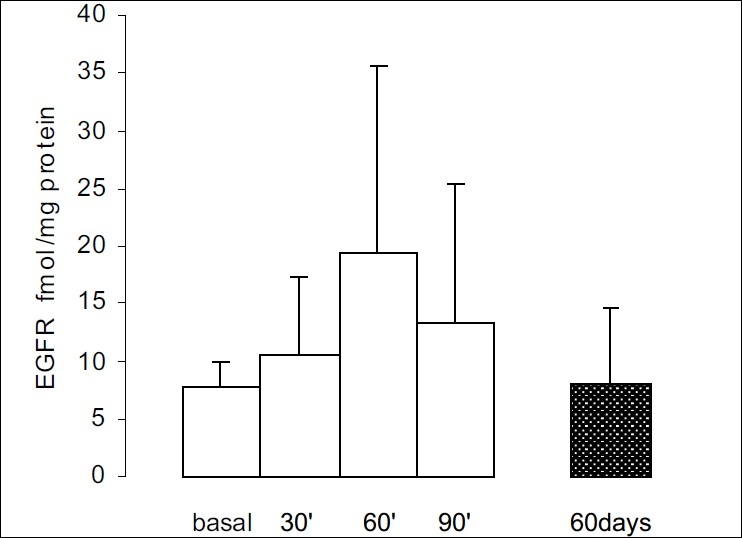
EGFR expression in the tumor membrane preparation (time course). Whereas 60 min after goserelin administration the mean EGFR expression in tumor membranes was higher than the basal values, the differences were not statistically significant (*P* < 0.49). Each bar represents the mean ± SEM and the values were expressed as fmol/mg of the total protein concentration.

In the isolated tumor membranes (n = 10), the mean basal expression of EGFR (BT) was 7.6±1.9 fmol/mg protein, and 30 min after “bolus” administration of goserelin these levels increased to 10.5±6.8 fmol/mg, while after 60 min they reached 19.4±16.2 fmol/mg before dropping slightly to 13.3±11.9 fmol/mg after 90 min. Chronic treatment with goserelin did not produce a significant change in the expression of EGFR (8.0±6.6 fmol/mg, n = 10) with respect to the mean basal values (*P* < 0.49, Figure 6).

## DISCUSSION

Mammary tumors can be considered as hormone-dependent or hormone independent tumors based on their hormonal requirements for growth.[17] Estrogen receptor (ER) expression has been reported in N-nitroso-N-methylurea (NMU) induced rat mammary carcinomas[18,19] although numerous reports have shown that breast cancer that initially responds to hormone therapy often develops into a hormone-independent type.

Goserelin is a potent luteinizing hormone-releasing hormone (LH-RH) agonistic analog that can be used for the treatment of hormone responsive tumors.[10] Daily administration of LH-RH agonist analogs leads to pituitary desensitization and a decrease in gonadotrophin release, which in turn produces gonadal atrophy and a decrease in sex steroid secretion.[20] Goserelin has also been shown to be effective against DMBA (dimethylbenzanthracene) – induced rat mammary tumors[21,22] and we previously observed an important regression in NMU tumor development after chronic goserelin treatment. This effect was associated with the inhibition of prolactin (PRL) expression, as well as the inhibition of TNF-alpha and nitric oxide (NO).[14]

After exposure to NMU, Copenhagen rats developed multiple microscopic nodules regardless of the treatment regime employed, and all these nodules were associated with the major blood vessels of the mammary glands.[23] When the expression of vascular endothelial growth factor (VEGF) was examined immunohistochemically,[2] VEGF was almost exclusively restricted to the tumor cells and no non-neoplastic breast tissue expressed VEGF. An increase in nitric oxide synthase activity was observed in VEGF positive tumors that reverted to basal levels following tetracycline-mediated suppression of VEGF.[24]

In the present “*in vivo*” study the expression of VEGF in NMU-induced rat mammary tumors differed, suggesting that VEGF expression reflects the heterogeneity in the NMU-induced-mammary tumors. Moreover, VEGF expression in plasma was affected by goserelin administration. VEGF expression was higher in rats with tumors than in healthy rats and the initial hyperstimulation by goserelin induced an increase in VEGF expression. Different molecules may interact to coordinate VEGF expression and it is known that one initial effect of goserelin is to stimulate gonadotrophin release. In addition, long-term goserelin treatment blocks the hypophysis and consequently, we observe a decrease in the VEGF expression.

We assayed VEGF expression in both plasma and in tissue homogenates to compare the variations in situ and we found a statistically significant increase of VEGF expression post-goserelin administration. When the time course of VEGF release “*in vivo*” was analyzed the initial increase in VEGF induced by goserelin was reverted during chronic treatment. In addition, there was association with regression in tumor size in all the rats after long-term treatment. The mean VEGF expression in plasma was lower in healthy rats than in rats with NMU-induced tumors, as reported previously.[25] Indeed, in female rats hyperstimulated with gonadotropins, VEGF and VEGFR-2 were over-expressed and this effect was associated with an increase in vascular permeability in the ovaries during acute exposure. However, GnRH-a (agonistic analog) treatment for 2 days in hyperstimulated rats produces a decrease in VEGF expression and that of its receptor (VEGFR), preventing ovarian hyperstimulation syndrome.[26]

The interaction between the VEGF and epidermal growth factor receptor (EGFR) pathways has been studied extensively[27] and a relationship between these growth factors clearly exists in solid tumors. Several studies using a small molecule tyrosine inhibitor of EGFR (e.g., gefitinib), or combined inhibitory therapy, demonstrated that VEGF is up-regulated by EGFR over-expression.[28,29]

The expression of LHRH receptors in tumors is associated with activation of the human EGFR and thus, activated EGFR phosphorylates, a 60kD protein that corresponds to the LHRH receptor.[30] Moreover, in colon adenocarcinoma LHRH binding sites other than the pituitary LHRH receptor may be involved in binding LHRH analogs.[31] Indeed, LHRH receptors are expressed more strongly in tumors than in normal cells.[32,33] These results suggest that LHRH analogs may bind to the LHRH receptors present in the tumor cells.

We found that EGFR expression was maintained at similar levels in the plasma and in the mammary tumors after goserelin administration, both during acute and chronic treatment. It should be noted that the ELISA method measures the total number of bound EGFR receptors and that the sensitivity of the method must be taken into consideration. Nevertheless these results are in agreement with the constant expression of EGFR in all tumors from both intact and ovariectomized Sprague-Dawley rats.[19]

In summary, NMU-induced rat mammary tumors regressed dramatically after goserelin treatment. VEGF expression was higher in NMU induced-mammary rats than in healthy rats, as its expression was modified in plasma and in tumor tissue by goserelin acetate. These results suggest that goserelin may provoke an increase in VEGF expression in the short term, whereas long-term treatment down-regulates VEGF. These results may be helpful to understand some mechanisms of tumor regression by goserelin and its possible direct or indirect effect on the angiogenesis.[14]

Unexpectedly, EGFR expression was not affected by goserelin treatment. To our knowledge, LHRH agonists should drop EGFR receptor numbers by different pathways; one would be preventing EGFR-mediated tumor growth through a PKC pathway.[34] However, we have to consider that the immunoassay used in this study assesses the total number of receptors and the results obtained by us could mask or not evidence the real number of active or functioning receptors. Future research studies may be addressed to solve this problem, which would make these findings clinically relevant.

**P.D:** This study was partially presented (oral communication) in the I World Cancer Congress, in Shanghai (China) 2008.

## AUTHOR'S PROFILE

**Irene Vegh, M.D. Ph.D.**

Born in Debrecen (Hungary). Upon her medical degree in National University of Cordoba in 1971 (Argentina), she join the Medical Faculty at Oncology Institute “AH Roffo” of National University of Buenos Aires, Argentina in 1972. This led to the development of antiestrogen treatment with clomiphene citrate and prolactin regulation and response in breast cancer patient. In 1988 she joined the Research Centre of Hospital Universitario 12 de Octubre, Madrid, Spain, where she upon completion of her doctorate in Internal Medicine – Universidad Complutense de Madrid, actually she is working in the same Research Centre as Senior Researcher. She was authored in several publications, and received many awards. Dr. Vegh obtained a personal Spanish Industrial Patent on Tissue Biomarkers Determinations (P -9401877, 1997), and an Intellectual Patent (1994). Concurrently, Dr. Vegh has applied various studies on predictive value of different biomarkers, allelic alterations and its prognostic value in colorectal cancer, non-small cell lung cancer, esophagus cancer. Actually she is working on carcinogenesis based in NMU- induced mammary tumors, their hormonal regulation, and combining basic with translational cancer research.
